# Is ‘Victim-Survivor’ Our Imperfect Alternative to Describing People with Lived Experience of Sexual Violence? A Feminist Symbolic Interactionist Analysis, Considering How Ethnicity, Gender, and Disability Interact with Language Choice

**DOI:** 10.1177/10778012251338454

**Published:** 2025-04-30

**Authors:** Laura Jane Bower

**Affiliations:** 3057 Department of Sociology, Durham University, Durham, UK

**Keywords:** victim, survivor, victim-survivor, sexual violence, symbolic interactionism

## Abstract

Feminist violence and abuse literature is caught in the grips of a debate surrounding the most appropriate language to describe people with lived experiences of sexual violence. This article offers a theoretical tracing of the history of the normative framings of “victim” and “survivor,” and the emerging alternative “victim-survivor,” through a symbolic interactionist lens. Given that both “victim” and “survivor” labels hold distinct disadvantages in isolation, particularly among the survivor discourse for ethnic minority and disabled and male victim/survivors, “victim-survivor” offers an alternative, in a similar fashion to LGBTQ+, affording flexibility for victim/survivors to occupy a multi-dimensional form of identity.

Amidst violence and abuse literature, there is an ongoing debate around what is the most appropriate language to describe people who have lived experience of sexual violence (O’Shea et al., 2024; Warner, 2023; Williamson, 2023). Individuals can only use the language available (Tumminio Hansen, 2020), where the most widespread labels within society appear to be “victim” and/ or “survivor” (Jordan, 2013; Setia et al., 2020). “Victim” is still considered the normative framing across academia, broader policy, and outside contexts (Hockett & Saucier, 2015). However, due to its more positive connotations, there is a contemporary preference for “survivor” within feminist conceptualizations (Dunn, 2005). Alternatives exist, albeit as fringe terminology, such as “thriver” and “overcomer” (Ben-David, 2020), or “victim-survivor” (LaFleur, 2024). These are relatively new terms, seldom used by victims, and rarely seen outside academia, policy and practice (Bower, 2024).

Although far from perfect, when viewed as an umbrella term to encapsulate the spectrum of experiences and identities of people with lived experiences of sexual violence, “victim-survivor” allows for flexibility and minimizes the reinforcement of the victim/survivor hierarchy, reducing the likelihood of devaluing disabled, ethnic minority and male victim/survivors. The offering of “victim-survivor” as the alternative in feminist literature has been rarely considered in scholarship examining the victim/survivor binary, particularly from a micro-constructivist symbolic interactionist lens, despite a recent push for usage in feminist discourse (Jean-Charles, 2014; O'Neill, 2018).

From a micro-level constructivist lens, “victim” and “survivor” are stigmatized identities inhabited and performed by people (Boyle & McKinzie, 2015). Identity refers to a

set of meanings that define who one is when one is an occupant of a particular role in society, a member of a particular group, or claims particular characteristics that identify him or her as a unique person. (Stets & Burke, 2000, p. 3)

Classical symbolic interactionism has offered that meaning-making is not just a set identity or group of characteristics; instead, identity is a reflexive process (Boyle, 2016). Language is also highly ambiguous due to the lack of universal definitions of “victim” and “survivor,” and the multi-dimensional ways victims/survivors use language (Leisenring, 2006). Studies have found that some people reject all labels as they wish to prevent their victimization experiences from being incorporated or given significance in their own identities (Williamson & Serna, 2018). Subsequently, caution may be expressed in being labeled at all with identity markers explicitly bound to abuse, due to fear that victimization will be given primacy in their identity (Ovenden, 2012). As all identity markers attached to sexual violence are inherently stigmatized (victim, survivor, victim-survivor, etc.), a framework that offers the conceptual power to understand stigmatization and identity, like symbolic interactionism, becomes vital.

In what follows, I examine the origins of the victim/survivor binary utilizing a symbolic interactionist theoretical lens to unlock cultural constructions of victim/survivorhood, specifically societal perceptions of labels and self-labeling of people with experience of sexual violence. To achieve this, existing literature exploring violence labels will be traced using symbolic interactionism as a theoretical lens to refocus and shift our understanding of key identity scholarship. Next, I position this amidst the normative use of the label “victim” within feminist literature and the growing advocacy for the term “survivor” amidst feminist scholarship and activist circles in the 1970s and 1980s. Furthermore, I ground this within the false classification of the victim-/survivor binary, examining how the “journey” metaphor held considerable consequences for victim/survivor labeling and how this shaped the meanings of victimhood and survivorship.

## Theoretical Positionality

Symbolic interactionism provides a contemporary framework for understanding identity, particularly re-examining how stigmatization fosters identity meaning for victims and the impacts of violence upon a person's sense of self (Boyle, 2016). Our sense of self is shaped and developed through interactions and communications with others (Blumer, 1969; Mead, 1934), so symbolic interactionists are particularly interested in how people use language (Dunn, 2008). Since the 1990s, symbolic interactionism has been used to understand victim/survivor identity labels beyond language choices or static labels (Konradi, 2011). Language can be self-given and conferred upon people with lived experience of sexual violence (Dunn, 2005). A person deciding what label to use to describe themselves is framed as a complex decision, marked with social, political, and even cultural consequences (Parker & Mahlstedt, 2010). Complexity in self-labeling is due to “victim” and “survivor” labels lacking universal consensus on what they mean (Schwark & Bohner, 2019), and definitions frequently vary from culture to culture (Papendick & Bohner, 2017). Victim/survivor identification also does not exist outside modes of oppression, and very little attention has been given to the specific implications of labeling for marginalized people (Boyle & Rogers, 2020), particularly with disabilities (Larson, 2018). Therefore, while there may be some commonalities in construct, identity labels can be very individualistic (Williamson, 2023). Crucially, a symbolic interactionist framework recognizes that a person typically occupies multiple identities, often bound to social situations (Stets & Burke, 2000). These identities are socially constructed and performed (Goffman, 1959). As a result, symbolic interactionism aligns well with victim/survivor identity, which is highly complicated due to lived experience “resisting formulaic presentation” (Loseke, 2001, p. 108).

Several studies have found that victims rarely identify with just one term throughout their post-victimization recovery. After interviewing victims of rape, Thompson (2000) found that some people identified with elements of both victimhood and survivorship but did not entirely claim either identity. Similarly, after interviewing victims, Leisenring (2006, p. 313) examined how victims hold multiple, sometimes even paradoxical or contradictory views of victim/survivorship. The victim label, especially, was often “sites of contestation and negotiation” (Leisenring, 2006, p. 313). Dunn (2005) noted that survivors sometimes used terms simultaneously, although participants still framed them as opposing. Dunn's (2005) findings further emphasize the paradoxical nature, suggesting identities are rooted within overly simplistic dichotomies.

## Labels and Cultural Constructions

Identity labels are not merely language but notions with influential representations of the individuals or groups attached to the label (Boyle & Rogers, 2020; O'Shea et al., 2024). As these representations are socially salient, they can result in those labeled, internalizing the representations explicitly bound to the label (Dunn, 2005; Leisenring, 2006). Thus, the label could become part of that individual's identity (Boyle & Clay-Warner, 2018; Williamson & Serna, 2018). Consequently, schemas and attached behaviors stemming from labels can be reinforced within an individual (Williamson, 2023), despite the behaviors being viewed as unfavorable or stigmatized (Boyle, 2016; Loseke, 2001). Neither term should be viewed as a monolithic concept; identity labels are discursive (Boyle & McKinzie, 2015). Therefore, symbolic interactionism centralizes the societal processes of discovering how someone becomes viewed as a victim or survivor.

While exploring the emotional management of victims of rape during the court process, Konradi (2011) felt that under a symbolic interactionist lens, identities are performed through a complicated process of emotional and impression management. Due to this stigma management, the choice of labeling oneself is explicitly affected by the cultural constructions of victims and survivors. Holstein and Miller (1990, p. 104) asserted that victims are “interactionally constituted” since the “meaning of objects does not inhere in objects, but is conferred upon them as they are interpreted, organized and represented through social interaction.” Central to this argument is the idea that no one is inherently or intrinsically a victim; instead, victim status is “conferred upon them” (Holstein & Miller, 1997, p. 26). When individuals’ sense of self diverges from normative societal expectations or their identity is regarded as possessing stigmatizing traits, they may embark on repair work (Dunn, 2005; Goffman, 1963). For instance, a victim may view their identity as damaged following a sexual assault (LaFleur, 2024). To distance themselves from the stigma associated with victimization, a person may embark on a process of impression management to separate their sense of self from the stigmatized identity aspect (Boyle, 2016; Dunn, 2005). We might even see this as renovating their sense of self (Draucker et al., 2009), perhaps adopting a survivor identity to signify their ability to cope with their victimization (Loney-Howes, 2018).

### Victim/Survivors’ Language Preferences

It is difficult to establish whether victims/survivors themselves prefer one term over the other, especially whether this is a strong preference. Studies focused primarily on self-labels tend to find a preference in self-labeling for the term “survivor” (Boyle & Clay-Warner, 2018; Johnson & Lewis, 2023; Williamson, 2023; Williamson & Serna, 2018). However, whether participants prefer the “exclusive” use of one or more labels matters in context when breaking this down. For example, Boyle and Rogers (2020) survey of sexual assault victims, including both men and women, found that while more participants preferred the survivor label exclusively (25%) over victims (11%), a higher number of participants expressed that they used both labels overall (44%). Thus, support for the survivor label is not always as strong as it seems. Moreover, Graham et al. (2021), in their survey of 375 college students who had experienced sexual assault, found most respondents (46.4%) preferred alternative labels to either victim or survivor (“other”). However, this does not necessarily support the suggestion of an alternative term, as respondents were only given the options of “victim” and “survivor” (Johnson & LaPlante, 2023). Moreover, no study to date solely focused on self-labeling following sexual assault or violence has given participants an option for “victim-survivor,” even though the term has been around since the turn of the twenty-first century (Bower, 2024).

A key issue with research examining self-labeling preferences is that some studies offer valuable insights for labeling. However, their primary focus is centred elsewhere, thus only giving us partial insight. Khan et al. (2018) conducted a mixed-methods study with an ethnographic component that only examined the “survivor” label and why participants rejected it, primarily focused on reasons for lack of self-acknowledgement of victim status and why sexual violence was not reported. In contrast, Phillips and Daniluk (2004) found that participants who had experienced child sex abuse viewed a survivor identity as a core part of their healing journey during counseling. However, their research did not specifically explore feelings of the victim label; in contrast, the primary focus was on self-definition in identity rather than the perceptions of identity labels. Identity-label studies also rarely consider the impact of various activist movements that form the context for the popularization of ‘survivors’ in feminist research (Warner, 2023). Minimal research has also been conducted with professionals working with victims who have experienced sexual violence (Bower, 2024), who may occupy a dual identity (Anderson & Overby, 2020). Moreover, nuance is needed in considering the victimization events of the people sampled, as there are distinctions between the experiences of sexual violence. These differences may reflect varying attitudes toward labels but have not been fully explored. Studies tend to concentrate on one type of victimization event rather than considering divergences or other forms of violence against women. With such a wide range of victimization experiences, a lack of consideration for the social movement context, and the intersecting experiences of victim/survivors, at times, identity-label studies lack generalizability beyond their scope.

## Historical Perspectives of the “Victim” Label

The “victim” label is arguably the most used historical term (Ben-David, 2020) and has a long-established history in victimization literature (Schwark & Bohner, 2019). “Victim” is often conceptualized as the first identity label that may be embraced or inflicted upon people after a victimization event (Boyle & Walker, 2016). The term “victim” is typically considered unfavorable due to their lack of agency (Rock, 2004), or powerlessness (Best, 1997). However, victims are also often perceived as innocent due to their apparent vulnerability from subordination (Holstein & Miller, 1990), or their blamelessness in terms of rape myths of precipitating their victimization (Dunn, 2005).

In their systematic review of rape-related literature, Hockett and Saucier (2015) found that “victim” is the normative label used, despite suggestions that there is a growing preference for the survivor label. Of course, it must be acknowledged that empirical research considering victim/survivor language is still very much predominantly being conducted with samples in the United Kingdom and North America, and the term “survivor” is utilized within research with Global South communities, such as formerly colonized countries like Kenya and Guatemala (Thomas et al., 2022). Furthermore, it can be incredibly tricky not to conflate distinctive terms about specific acts of violence; Hockett and Saucier (2015) acknowledge that there is far more literature concerning the experiences of language within “sexual assault” rather than “rape.” Sexual assault is more of an umbrella term due to its breadth (Leung, 2017), referring to a sexual act “committed or attempted by another person without freely given consent of the victim or against someone unable to consent or refuse” (Basile et al., 2014, p. 11). In contrast, “rape” is a distinctive subcomponent (Buchwald et al., 1993). However, it is hard to define due to subtle differences in the usage of the term across academia, policy, practice, and everyday culture (Brown et al., 2023). Framings of rape globally tend to have three core aspects: the absence of consent, the use of force, and penetration (Decker & Baroni, 2011). As both are distinctive experiences, we must be cautious in generalizing identity experiences beyond the research focus category, particularly as the two terms are often used interchangeably inaccurately (Hall & Flannery, 1984).

### Victim as a Negative Label

Studies examining the connotations toward the “victim” label find the label overwhelmingly associated with negative societal attributes (O'Shea et al., 2024; Schwark & Bohner, 2019). People who have experienced sexual violence exist within a broader neo-liberal society that values individual strength (Garland et al., 2006), so may show hesitancy in occupying the victim label due to its overwhelming connotations (Rock, 2004). Victimhood is something that people are encouraged to move away from, as victims are viewed as exhibiting learned helplessness (Seligman, 1991), when a person takes no action when encountering obstacles or setbacks (Ben-David, 2020).

Learned helplessness is often conceptualized as a failure to cope with a traumatic circumstance (Dussich & Jacobsen, 1981). Illuminating our understanding of the cultural expectation that a person should refuse to be a victim, “victim” itself no longer encapsulates a person's position within the patriarchal power structures that abuse occurs in, but reflects a personality marked by “incomplete personhood” (Haraway, 1997, p. 65). Therefore, “discourses of personal responsibility are consistently embedded in cultural narratives of pain” (Patsavas, 2014, p. 209). However, other structural conditions or forms of oppression that contribute to violence and abuse are ignored, as victims are positioned as needing to assume the mantle of responsibility for their recovery (Larson, 2018).

### Modes of Oppression

Language usage, of course, does not exist in isolation from modes of oppression. Christie's (1986) conceptualization of the ideal victim illuminates how “true” victims must be perceived as playing no role in their victimization and demonstrating their innocence. The key positive of this trope is that “proper victims” can be offered sympathy due to their lack of culpability in their actions (Loseke & Best, 2003). D’Souza (1991, p. 242) dubs this as a “quest” for “the moral capital of victimhood,” a quest more cumbersome for ethnic minorities who are more likely to have their victim status called into question. Of course, all victims of sexual assault are exposed to some form of blame (Boyle & Rogers, 2020), but white female victims are often framed as being more likely to be recognized in cultural narratives (Christie, 1986).

Asian women have been subjected to extreme fetishization, resulting in significant sanctions on their bodies and sexual behavior (Woan, 2008). Latinx and Black women are also often subjected to much deeper condemnation and sanctions upon their behavior and appearance within discourses of victim-blaming (Spohn et al., 2001). Black women's bodies have been hypersexualized and legitimizing their victimization due to deeply embedded constructions bound to colonialism and slavery (Zounlome et al., 2019); frequently subjected to cultural narratives that blame them as “asking for it” (Littleton & Dodd, 2016). Latinx women have also been attached to constructions of engaging in risky behavior amidst sexual and substance-taking behavior (Slakoff & Brennan, 2019). Thus, this explicit bounding of innocence to victimhood highlights the subsequent distortions of justice within certain ethnic groups surrounding their actions (Convery, 2006).

There has been little incorporation of examining victim/survivor identity labels from an intersectional standpoint (Bower, 2024). However, some studies have examined the impact of gender (Setia et al., 2020). Boyle and Rogers (2020) surveyed over 1,000 college students to examine the interaction of class, race, and gender in shaping attitudes toward victim/survivor labeling. While they did not explicitly utilize intersectionality as a theoretical framework and most of their sample was white (*n* = 83.4%), there was some attempt to consider how overlapping identities could shape identification that would align with intersectional conceptual ideas, particularly the experiences of women of color. Intersectionality illuminates how multiple forms of oppression can intersect or overlap (Crenshaw, 1991), where these intersections have perpetuated the further marginalization of invisible groups (Hockett & Saucier, 2015). For example, we know that sexual violence disproportionately affects women of color (Curtis et al., 2023; Decker et al., 2019), where minority women are frequently denied the “legitimacy of victimhood” (Wallace et al., 2024, p. 6). This might also shape how they view victim/survivor identity labels, given their subjugation to different cultural constructions. However, without an intersectional lens, we struggle to fully appreciate how individuals may choose identity labels and how their experiences may vary based on multiple identity markers. Naturally, this is a real apparent blind spot in victim/survivor labeling literature, as we fail to fully capture and appreciate the authentic experiences of victims and survivors, only understanding the experience in a one-dimensional manner.

## Historical Perspectives of the Survivor Label

Feminist circles in the 1970s and 1980s began challenging dominant cultural narratives of victim precipitation and blaming (Gordon & Riger, 1991). Rape narratives shifted quickly from simply conceptualizing rape as unwanted sex to it being positioned as “a permanently devastating experience” (Chasteen, 2001, p. 135), where women's lives were viewed as permanently ruined by sexual assault within the media (Gavey & Schmidt, 2011). Amidst this backdrop, Barry (1979, p. 39) offered “survivor” as a viable alternative to the victim label, as “surviving is the other side of being a victim. It involves will, action, and initiative on the victim's part.” Similarly, Kelly (1988) noted that while rape could have a horrendous impact on a woman's life, it should be constructed as a survivable crime, where victimization did not have to take primacy over a victim's identity. Here, we see a direct challenge to the way that sexual victimization had been positioned as a rupture of a person's identity. This rupture could trigger a noticeable “association of the true self” (Kline, 2007, p. 737). As a result, this is quite an extreme viewpoint to suggest sexual violence always results in an identity rupture, as sexual assault does not always impact all aspects of a person's identity (Fater & Mullaney, 2000).

Most conceptions within feminist violence and abuse literature do tend to frame “survivors” as distinctive from “victims” (Convery, 2006). While “victim” concentrates on the victimization event, “survivor” focuses on what happened in the aftermath (Boyle & Clay-Warner, 2018). Therefore, survivors are often constructed to showcase strength post-victimization (Hockett & Saucier, 2015; Leisenring, 2006; Thompson, 2000).

### Strength Within the Survivor Label

A neo-liberal society is individualistic and “prizes strength and personal responsibility” (Loseke, 1999, p. 14), so “survivor” has more cultural value than the devalued victim label (Leisenring, 2006). Survivors are positioned as active agents moving toward recovery and resisting patriarchal violence (Boyle & Rogers, 2020). Although this is a damaging cultural narrative, by ignoring the social systems that contribute to, allow, or condone victimization, we inadvertently enable the oppression and reproduction of rape culture by rewarding survivors (O'Shea et al., 2024).

In tandem, it can have specific implications for black women (Boyle & Rogers, 2020). There is a dominant cultural narrative of “the strong black woman” that has perpetuated the idea that black women should prioritize racial struggles above their well-being, constructing archetypal modern women today as able to cope with adversity (Collins, 2004; Watson & Hunter, 2016). Here, we see that “strength” can also result in evident “sacrifice” for black women (Wyatt, 2008). In addition, Asian women are also subjected to dominant cultural narratives of the expectation that they should be strong and tough (Im et al., 2011). Moreover, Latinx women have been frequently subjected to culturally gendered expectations grounded in marianismo, stemming from Spanish colonial ideas of women being self-sacrificing, encouraging women to remain silent about their pain (Chavez-Dueñas & Adames, 2021). Thus, some women of color face distinctive pressures in adopting or resisting the survivor label that has unique implications that have truly yet to be explored in identity-label literature.

Although black women are more commonly discussed in terms of victim/survivor labeling, culturally pervasive narratives of an expectation of strength affect males as well. Male victim/survivor label identification has been chronically underexplored in violence and abuse literature (Bower, 2024). However, we can see male victim/survivors subjected to quite dominant cultural narratives. Men are subjected to gender norms that expect them to be strong and unemotional (Donne et al., 2018), but experiencing victimization contradicts hegemonic masculinity (Petersson & Plantin, 2019). Men are concerned about being labeled weak (Sable et al., 2006). They may not want to be seen as victims of sexual assault because of fears of being perceived as damaged (Kwon et al., 2007), as it directly contradicts the stereotypical image of a strong and silent man. Thus, victim status comes at “the cost of manhood” (Tryggvadottir et al., 2019, p. 1001). Again, although these discourses have been chronically ignored in studies examining victim/survivor identification, it must be at least conceptualized that all these surrounding discourses could potentially impact how male or ethnic minority victims/survivors may be concerned about perceptions surrounding them, as they are forced to navigate language-identification decisions within these dominant cultural narratives.

### The Problem of Language in Sexual Violence: Current Perceptions

While “victim” and “survivor” labels are positioned as central identities, they are typically conceptualized as having distinctive connotations underpinning them (Van Dijk, 2009), polar opposite or even in binary categories (Boyle & Rogers, 2020; Dunn, 2005; Kelly et al., 1996). Victimhood cannot be viewed as a solely negative identity, as there are clear benefits to claiming victim status. Victims may need to claim victim status to be recognized as the injured party within the criminal justice system, particularly where victim status is often questioned, such as intimate partner violence (Dunn, 2010). Claiming victim status can help escape being viewed as deviant (Boyle & McKinzie, 2015). It may make the person be afforded sympathy and support (Leisenring, 2006), particularly in areas of sexual violence where it may be desirable to be viewed as powerless, such as domestic abuse (Dunn, 2005). In parallel fashion, the survivor label did not receive mainstream support from feminist scholars and activists (Lamb, 1996), so asserting that there was a complete paradigm shift would be simplistic. Arielle (2016) raised concerns about the damaging stereotypes an alternative label could create, such as the frequent binding of “survivor” with agency (Schwark & Bohner, 2019), achieving little to further our understanding of victims’ experiences (Gavey, 1999).

Agentic actors are viewed as owning their actions (Naples, 2003). While during the victimization experience, the victim/survivor is positioned as having no agency, thus meeting the threshold of a non-consensual sexual act. However, agency can be expressed in the aftermath or post-assault (Jean-Charles, 2014). “Survivor labels paint a picture of agentic individuals who do not passively experience abuse” (Williamson & Serna, 2018, p. 670). Thus, agency is often viewed through the lens of recovery within the survivor discourse, where survivors are positioned as taking active steps toward healing. For instance, Sweet (2021) framed the performance of survivorhood as a mechanism to allow women who have experienced intimate partner violence to receive access to support. However, agency within the survivor label has been positioned as a “hollow victory” (Dunn, 2001, p. 309), as it reconfigures responsibility onto the individuals themselves, drawing attention away from the damaging patriarchal social structures that force victims to hold agency.

We see this quite clear victim/survivor dichotomy (Boyle & Rogers, 2020), namely, a discursive dichotomy (Picart, 2003), resulting from positioning victims and survivors at opposite ends of the continuum based on either agency (Dunn, 2005) or strength (Miller, 2018). It was a division on the grounds of agency between the “passive victim” and the “active survivor” (Alcoff & Gray, 1993, p. 261). The divide was seemingly unnecessary (Convery, 2006), where positioning victims as inadequate at coping with adversity creates a hierarchy among survivors (Elias, 1985). “Survivors” are constructed as the “fittest” victims due to their ability to cope with adversity, and nonsurvivors, in other words, victims, should be “deselected” (Convery, 2006, p. 240). It positions survivors as “entirely distinct sets of individuals,” possessing some inherent qualities victims do not have (Convery, 2006, p. 241). This further reinforces a hierarchy among victims/survivors (Boyle & Rogers, 2020), devaluing those who fail to measure up to the standard of survivorhood. Tension can also arise when considering who is deserving of sympathy and support, holding particular consequences for marginalized victims/survivors, such as LGBTQ+, ethnic minorities and Indigenous women (O'Shea et al., 2024), those with disabilities (Larson, 2018), especially intersecting identities.

## Journey From Victim to Survivor

A journey metaphor is commonly deployed to explain this transition from victim to survivor, wherein victims later adopt the term “survivor” (Williamson, 2023, p. 15). This journey is usually considered therapeutic, where victims receive external support to move past their trauma (Gavey & Schmidt, 2011) and hold a more positive self-identity (Williamson & Serna, 2018). Thus, it is often described as a linear progression (Jordan, 2013). However, linear progression is somewhat controversial, as “linear” suggests upward progression and fails to represent the many setbacks victims often encounter (Boyle & Rogers, 2020). In addition, the idea of complete recovery is also often conceptualized as unrepresentative of experiences of trauma (Leisenring, 2006).

### Strength Within the Journey Metaphor

Although “victim resilience” is an existing conceptual idea (Bonanno, 2005), resilience is much more commonly tied to survivorship (Dunn, 2005). A key part of resilience is this idea of unexpected inner strength that survivors can draw upon despite extremely traumatic experiences (Van Dijk, 2009), where they can continue to live (Papendick & Bohner, 2017), perhaps even thrive (Ben-David, 2020). Strength and resilience become a particular issue when considering cultural narratives surrounding disability. We start creeping into neo-liberalist theorizations where both trauma and mental illness become framed as barriers a victim must overcome (Bower, 2023). Linton (1998, p. 18) expressed concern with language rooted in the need for people with disabilities to “overcome” their impairment; as the suggestions create stigma among people who are unable to do so successfully (Linton, 1998; Mitchell & Snyder, 2001). Overcoming subsequently reproduces a climate of “compulsory able-bodiedness” that people with disabilities fall short of (McRuer, 2006).

Larson (2018, p. 689) observed that as a survivor identity is prized and a victim identity is devalued due to the emphasis placed upon the survivor label of strength and resilience, creating “compulsory survivorship.” There is an expectation that survivors must become free from any trauma-related disabilities, including post-traumatic stress disorder (Bower, 2023; Larson, 2018). Thus, under compulsory survivorship created through prizing survivors' resilience, victims are conceptualized as unable to maintain normal mental functioning and thus are “socially beneath their able-bodied counterparts” (Larson, 2018, p. 689). Sexual assault is usually considered one of the most serious forms of trauma (Swanson & Szymanski, 2020). Thus, many victims/survivors may struggle to live up to high expectations of strength and resilience.

Convery (2006) adopted a metaphor from social Darwinism to explain this as the survival of the fittest, where disabled victims/survivors are completely devalued and delegitimized due to failing to negate their disability. This marking of survivors as “distinctive” sets of individuals fails to consider the intersecting modes of oppression that may act as a barrier to recovery, as the survivor discourse completely neglects that not all victims are on a level playing field to transition. After all, “victims need more than just their ‘selves’ to become survivors” (Orgad, 2009, p. 153); access to therapeutic support or external resources varies significantly.

## The Victim/Survivor Binary

At the turn of the twenty-first century, criticisms began to emerge of the “survivor” label (Young & Maguire, 2003) and the framing of “victim” and “survivor” as two mutually exclusive terms or distinct categories (Boyle, 2016). Due to this binary framing (Dunn, 2005), people were caught in a “VictimSurvivor Paradox,” forced to choose and sacrifice the benefits the alternative label brought (Thompson, 2000, p. 328). “Survivor” may grant agency but bring the expectation of recovery and could lead to the denial of social or therapeutic support (Young & Maguire, 2003). Similarly, “victim” can act as a pathway for support as it allows “the awfulness of rape” to “be appreciated” (Thompson, 2000, p. 330) but victims could be viewed as weak or vulnerable (Leisenring, 2006). The paradox forces victims/survivors to either reduce their agency or minimize their trauma (Thompson, 2000); it is a “dilemma of conflicting consequences” to be navigated (Papendick & Bohner, 2017, p. 4).

Feminist scholarship has attempted to remove dichotomous divisions. Kelly et al. (1996) suggested a feminist praxis beyond viewing the two labels through simplified binaries, Bahar (2003) proposed dismantling constructions of victim identity with passivity, and Goodey (2005) felt there was no need for a division at all, laying the foreground for the adoption of “victim-survivor.” Even Barry's (1979, p. 39) famous attestation that “surviving is the other side of being a victim” might seem a strong advocacy for separating terms. In actuality, her arguments were more closely centred on addressing the culturally persuasive narratives that victims/survivors were subjected to, rather than offering a new linguistic term.

### Victim-Survivor: Current Conceptions

The emergence of “victim-survivor” and “victim/survivor” as alternatives is quite challenging to track (Boyle & Rogers, 2020). However, they began appearing in feminist literature in the mid-2000s (Jordan, 2013). For example, victim/survivor is often used as an “and/ or” term (for example, see Holland et al., 2021; Ovenden, 2012; Papendick & Bohner, 2017). Now, it must be stressed that although “victim-survivor” has remained a marginal term in feminist literature, and arguably still is today despite its increasing adoption, it has been in use since the late 1990s (such as Reed, 1995; Rozee & Koss, 2001). However, apart from LaFleur’s (2024) cultural cognitive review, very little consideration has been given regarding how “victim-survivor” should be used.

“Victim-survivor” itself should not be viewed as an alternative label that victim-survivors themselves may identify with. Instead, “victim-survivor” is an all-encompassing umbrella term to capture the diversity of identities of people with lived experiences of violence. The term can be used as a default language when the individual victim/survivor’s preferences are unknown. It moves away from binary construction where “victim” and “survivor” are seen as fixed, immutable categories, where a person can only identify with one. Instead, it allows language to traverse both and bridge the unnecessary gaps that binary constructions have created. Victim-survivor also allows for a category more in line with self-labeling literature. Furthermore, it breaks away from incessant tendencies of the binary approach to categorize people with lived experience of sexual violence in public narratives, despite this approach lacking compatibility with the actual lived experience of victims/survivors (Zerubavel, 1991).

We could think about victim-survivor as comparable to LGBTQ+ in terms of sexuality, where although the initials stand for specific identities, the term signifies others that are not explicitly listed (Thelwall et al., 2023). As an initialism, here the moniker “LBTQ+” serves as a broad term, aiming to capture a range of personal and sexual identities shaped by interactions, in an area where people use a wider variety of terms (Russell et al., 2023). The “+” functions to indicate all remaining gender identities and sexual orientations not represented by the earlier initials (Thelwall et al., 2023, pp. 2515–2516). Whether a plus is needed for victim-survivor becomes interesting, as there are alternative identity markers, for instance, “thriver” and “overcomer” (Ben-David, 2020). However, these are very much fringe terms in terms of mainstream usage by victims/survivors themselves (Bower, 2024). A “+” in victim/survivor labeling could be misinterpreted to reproduce a hierarchy, where instead of “in addition” as it functions in the LGBTQ+ community, it could be unintentionally positioning some identities as superior, such as the ones named vs those not represented.

LaFleur (2024) advocates strongly for “victim-survivor” over “victim/survivor,” as a slash suggests that the terms can be used synonymously or in conjunction. “Victim-survivor” “holds in tension their paradoxical (in)divisibility and (dis)continuity. It is an overt nod to liminality—the inter-, in-between” (LaFleur, 2024, p. 230). LaFleur's suggestion centres on breaking away from the narrative of transformation that identity labels have been plagued with, namely that a person must journey from “victim” to “survivor.” Instead, “victim-survivor” suggests a transition from “unvictim” to “victim-survivor,” allowing a person to take on an identity marker with sexual violence, but separating them from the profoundly entrenched connotations of both terms. So, a transition still exists, but the transition is rooted in the victimization event rather than post-violence work. The argument offers a powerful reconceptualization and moves away from the false dichotomies created by the victim/survivor binary. However, describing “victim-survivor” as a “nod to liminality” suggests some form of transition between the terms themselves, reproducing the transition LaFleur seeks to sidestep.

“Victim-survivor” does offer the most linguistic potential for avoiding a suggestion of a continuum, as “victim/survivor” suggests an evident ability to separate the two and suggests distinctive, distinguishable identities. Here, perhaps we come to the notion that “victim-survivor” is an imperfect alternative, but great caution is needed in how it is conceptualized, mainly centred around scholars' intentions. Adopting “victim-survivor” linguistically “lumps” the two terms together (LaFleur, 2024), but it serves far more than just a linguistic purpose. From a symbolic interactionist standpoint, the labels applied, claimed, and resisted by victims/survivors affect how they are perceived. Both terms have quite distinctive cultural constructions, or at least this is how the victim/survivor binary has constructed them. A hyphen can “lump” these two categories together and allow victims/survivors to transcend this supposed inter-categorical variability that the binary perpetuates and move away from framing victimhood as a more discredited identity. Particularly if victims/survivors themselves seem to use terms interchangeably upon occasion (such as Leisenring, 2006), thus lumping can have substantial benefits of dismantling the problematic binary and have the language used in academia be more in step with the ways victims/survivors themselves utilize labels.

By adopting “victim-survivor,” academia can ensure that despite negative connotations attached to “victim,” individuals still use it to describe their identity (Larson, 2018). It also allows victims/survivors to occupy both labels, as many victims/survivors occupy multiple identities throughout their recovery or depending on the situation (Leisenring, 2006) and acknowledges that victim status can be beneficial to claim in specific settings (Ben-David, 2020). Victim-survivor also offers the complexity needed to capture the contours of sexual violence, breaking away from the drawbacks of each term in isolation, especially for ethnic minority victims/survivors. As a broader umbrella term, it encapsulates how individuals may fluctuate in their identification (Hockett & Saucier, 2015).

By separating the two into distinctive identities, feminist literature is always going to reinforce the victim/survivor hierarchy, whether intentionally or not. This hierarchy has substantial consequences for devaluing disabled victims/survivors and ignoring the dominant cultural narrative affecting women of color and males mainly. Therefore, to avoid this hierarchy, the “victim-survivor” should not be viewed as a placeholder or two separate identities. Instead, “victim-survivor” should be constructed as an umbrella term to encapsulate the broad spectrum of identities of people with lived experiences of sexual assault. After all, a person can theoretically be “queer” and “demi-sexual,” while still using the term LBTQ+, the term serving as an umbrella reflects this even though one of these identities is not explicitly represented.

## Conclusion

Victim-survivor is hardly perfect, nor is it a normative framing across policy, practice and academia for people with lived experience of sexual assault (see Bower, 2024; Hockett & Saucier, 2015). However, given the overwhelmingly negative construction of the victim identity (Leisenring, 2006) and the hesitancy this has created within victims to adopt its usage (Rock, 2004), there are stark limitations to the victim label. Although it can allow for the seriousness of sexual violence to be appreciated (Thompson, 2000) and victims to be deemed innocent (Dunn, 2005). We can see key drawbacks with the other alternative, survivor, as while it may grant individuals agency (Alcoff & Gray, 1993), it can prevent survivors from accessing support (Young & Maguire, 2003). Survivorship, being explicitly bound with agency, also perpetuates a social Darwinist survival of the fittest rhetoric, suggesting that there is something distinctive within survivors that separates them from victims, reinforcing a key victim/survivor hierarchy (Convery, 2006). It also completely ignores the fact that survivorship is rarely in isolation down to the individual (Orgad, 2009), and marginalized victims/survivors are subjected to very dominant cultural narratives perpetuating strength, resilience and emotional blankness. Thus, the survivor label holds significant limitations through reinforcing the hierarchies to which disabled, male and ethnic minority survivors are subjected.

If “victim-survivor” is adopted and viewed as an umbrella term in a similar fashion to LGBTQ+, it can be a good alternative to the victim/survivor binary. Victim-survivor acknowledges the strengths and diffuses the limitations of both terms in isolation, minimizing the reproduction of the hierarchy. It also affords victims/survivors the flexibility to occupy a multidimensional form of identity, depending on their point in recovery and the situation (Leisenring, 2006). It may be an imperfect solution, given that it is still a relatively peripheral term, but it is a good solution. Thus, perhaps we are one step closer to silencing the victim/survivor binary for good after all.

## Acknowledgements

The author would like to thank Nicole Westmarland, Kelly Johnson, Sui-Ting Kong, Stephen Burrell, Fiona Vera-Gray and Ruth Lewis for their sincere support and feedback.
